# New Styptic

**Published:** 1853-04

**Authors:** 


### ARTICLE XIV.

### *New Styptic.*

Mr. B. W. Richardson drew the attention of the society to a new styptic which had lately been introduced to the notice of the profession by Signor Pagliare of Rome, and which had received an extensive notoriety, general as well as professional. The styptic had been alluded to in this country by the London correspondent of the *Provincial Medical and Surgical Journal.* The composition of it was as follows:—Gum benzoin, eight ounces; alum, a pound; water, ten pints. The alum and benzoin were boiled for eight hours in the water; fresh water being added to make up for loss by boiling. The supernatant liquor was the styptic in question, the virtues of which were said to be such that if one drop of it were added to a basin of blood, the whole would instantly coagulate. On reading this description of the styptic, Mr. Richardson thought it would be of great service to bring the matter to a fair trial; he had, he thought, done so, and he now would lay the results of his experiments before the society. The solution employed had been carefully made in the laboratory of the Messrs. Taylor Brothers, of Vere street. A small bottle of it was presented for the inspection of fellows. The experiments that had been performed, were made both on the human body and on inferior animals. The first had reference to freshly-drawn blood. Two basins of mixed blood, drawn rapidly from the neck of a sheep, were placed side by side in the open air, the day being rather cold; into one of the basins a few drops of the solution were dropped; at the end of two minutes the blood in both basins was firmly coagulated, that with which the solution had been mixed coagulating but three or four seconds at most, previous to the other; indeed, it required the closest observation to observe any difference. This experiment was repeated five times with the same results. On examining the blood in the basins after separation into clot and serum had taken place, those clots were found to be darkest to which the solution had been added. It was found, also, that the serum of that blood which had received none of the solution yielded on the application of heat, small white albuminous flakes, which did not happen with the serum of that blood which had received the styptic. Secondly, the ear of a living sheep was slit up from the base to the apex, and the solution was applied to the incised parts. The application certainly did produce considerable arrest to the hemorrhage, but not in a degree greater than would have been expected from the use of other powerful styptics. This experiment was repeated with similar results. In a patient belonging to Dr. Willis, of Barnes, in whom profuse hemorrhage was taking place from the rectum, the styptic solution was used as an injection, at the suggestion of the speaker. The hemorrhage was effectually checked. It was right, however, to state that the patient was at the same time taking gallic acid in liberal doses. Lastly, he (Mr. Richardson) had recently had an excellent opportunity of testing the effects of this new styptic in comparison with old ones. Six leeches had been applied to the chest of a child under his care, and the friends had summoned him in great haste to check the bleeding, which was most profuse. There were three points from which the blood flowed; to one of these points the new styptic was freely applied, to another a saturated solution of alum, and the third was touched over with lunar caustic. In all three instances the styptics were only partially successful. The caustic, however, was most efficient, the alum solution next so, and Pagliare's solution least of all. They were all tried as long as was compatible with safety, and, finally, pressure was used with perfect success. Mr. Richardson's opinion, therefore, was, that the styptic powers of the solution of Signor Pagliare had been much overrated. That it was a styptic he had no doubt, for it contained alum. He did not know whether in the mixture a chemical combination was formed betwixt the alum and the benzoic acid; but he thought not. It was, probably, a mere solution of alum containing benzoic acid.

